# Parents’ knowledge and socio-demographic determinants toward child’s restraint system use

**DOI:** 10.1186/s12887-023-04136-5

**Published:** 2023-06-22

**Authors:** Sepideh Harzand-Jadidi, Homayoun Sadeghi-Bazargani, Koen Ponnet, Milad Jamali-Dolatabad, Barbara Minuzzo, Amirreaza Kamrani, Mahdieh Abbasalizad-Farhangi, Fatemeh Bakhtari Aghdam, Leila Jahangiry

**Affiliations:** 1 Road Traffic Injury Research Center& Department of Health Education and Promotion, Tabriz University of Medical Sciences, Tabriz, Iran; 2 Faculty of Social Sciences, Imec-Mict-Ghent University, Ghent, Belgium; 3 Communities That Care Ltd, Parkville, VIC Australia; 4 Department of Health Education & Promotion, Tabriz University of Medical Sciences, Tabriz, Iran; 5 Department of Community Nutrition, Tabriz University of Medical Sciences, Tabriz, Iran

**Keywords:** Child Restraint System (CRS), Road traffic, Booster seat, Knowledge, Barriers, Iran

## Abstract

**Background:**

Lack of protection or improper protection, is one of the most important reasons of child passenger’s death and injury in traffic crashes. Based on what we see on the roads, Iranian children are unrestrained inside the car. The aim of this study was to investigate children restrained system (CRS) use rate, its socio-demographic determinants and parents’ knowledge toward CRS use among Iranian parents.

**Methods:**

Using multi-stage cluster sampling and direct in filed method of observation, the behavior of 700 children in cars was observed in the current cross-sectional study. Socio-demographic determinants and parents’ knowledge, toward using the CRS were evaluated using questionnaires. The study was performed from July to August 2019 in Tabriz city, northwestern Iran.

**Results:**

The rate of child safety seat (CSS) use was 15.1% CI 95%:(12.5%,18.0%), and the rate of booster use was 0.6%; CI 95%:(4.3%,8.0%). The majority of parents [e.g. 64.3%; CI 95%: (60.7%,67.9%)], had low knowledge about the use of CRS. The most important reasons for not using CRS was lack of laws and policies [e.g. 59.7%; CI 95%:(12.5%,18.0%)], lack of knowledge [e.g.59.6%; CI 95%:(57.9%, 63.3%)] and the high cost of CRS [e.g. 57.6%; CI 95%:(53.81%,61.2%)]. The most important predictors of not using CRS were the child's age, parental knowledge, and the socioeconomic status of the household (*p* < 0.05).

**Conclusions:**

Most children did not have CRS. The parents with higher education and those with higher socioeconomic status had higher rate of CRS use. Based on the low rate of CRS use and poor parental knowledge about it, education of parents toward boosters use and benefits of using CRS, enforcing mandatory laws and ploicies for CRS use in Iran, and allocation of government subsidies to low-income families for purchasing CRS are suggeted as essential strategies to increase CRS use.

**Supplementary Information:**

The online version contains supplementary material available at 10.1186/s12887-023-04136-5.

## Background

Motor vehicle crashes (MVCs) are the leading cause of death and injury for children under 15 years of age. Annually, 186,300 children die as a result of MVCs in the world (more than 500 children per day or one child every four minutes) [1, 2]. In some low income countries similar to Iran for example Vietnam, 2,000 children died per year or equivalent to 5 children died per day because of MCVs [1]. Vietnam’s Ministry of Health reported that child traffic accident fatality rate of Vietnam was about 20 deaths per 100,000 children, while the figure was 7.4 deaths per 100,000 children in South East Asia and 4.2 deaths per 100,000 children in Europe [1, 2].

It can be stated clearly that South East Asia children such as Iranian children are at the high risk of fatality when they participate in traffic. Children who do not die in MVCs are severely injured [1, 2]. Children’s physical characteristics such as having a large head compared to the body cause them to have more head injuries in accidents. Moreover, their smaller weight and size cause them to be ejected. These characteristics make child occupants more vulnerable [3]. The length of the period of children’s disability imposes a heavy financial and psychological burden on the family and society [4].

Study in Canada has shown that 398 children under the age of 14 died in traffic crashes between 2008 and 2012. In the United States, MVCs have been the leading cause of death for children under 13 [5], and about 100,000 people are killed in MVCs in China annually, 12% of whom are children [6]. MVCs are also ranked among the top five causes of injury and death among children under 14 years of age in the Victoria State of Australia [7] and the second most frequent cause of death in Indian children that was 9.4 percent [8].

In South Asia and Africa, the rate of disability and mortality of RTIs is 7.4 and 19.9 per 100,000 children, respectively. The mortality and disabilities attributable to road traffic crashes in Iran is considerably higher. In 2016 alone, road traffic injuries were linked to 35.6 (29.64–43.33) deaths per 100,000 children. Children aged 17 years or younger constituted a notable percentage of those injured in Iran, representing approximately 14% of those receiving RTIs [9]. Out of all children killed in accidents, 36% are passengers [2]. In Turkey, neighbor of Iran, among patients under 16 who were admitted to an emergency department of a training and research hospital was determined that the cases who were dead constitute 0.7% of all cases and the majority of them occurred due to in-vehicle traffic accident. Also 56% of them injured head and neck region of the body [10]. Also Ozturk's study in Turkey showed among the children who died from RTIs, 6.65% were drivers, 41.31% pedestrians and 52.04% passengers [11]. Based on the studies in Iran, MVCs are the second cause of death, and more than 17,000 people die as a result of MVCs annually [4].

There is no accurate information about child death statistics in Iran, but seven children die in MVCs every day according to police reports [12]. Most of the MVCs and injuries in children are due to not using the child restraint system (CRS) or using it improperly [3]. The use of CRS which is appropriate for a child’s age and weight can reduce children's fatality rate [13]. Statistics show that, in MVCs all over the world, about half of the victims aged 4–8 years did not use CRS [14]. The World Health Organization (WHO) considers it necessary for children to use the CRS up to the age of 10 or a height of 135 cm [2]. Since almost all children are transported by car, therefore, it is important for them to be in proper restraint systems [15]. Thirty-three countries enforce laws on CRS use for children [16]. A child safety seat (CSS) can reduce infant fatality by 70% and the fatality of children under four by 54–80%. Moreover, the use of a booster (seats for children aged over four) reduces the risk of severe injuries in children by 59% [17]. The goal of CSS and the booster is to reduce MVC fatalities and injuries in children under 12. As car seat belts are designed for people over 4 feet and 9 inches tall, it is the role of the booster to lift children and teens to improve seat belt performance [3]. The probability of injury in children aged 4–7 years, who use seat belts with a booster, is 59% less than when they use only the seat belt [14]. Children who use only seat belts are at high risk, and the probability of serious injury for these children increases to 153% [5]. The risk of early use of seat belts for children includes head, spinal column, lung, and abdominal injuries. Therefore, children under the height of 135 cm must use CSS or a booster [18, 19].

In addition to using a restrained system among children, other activities are needed including child passenger restraint laws, car and booster seat distribution plus education programs, community-wide information plus enhanced enforcement campaigns [20]. Also Types of interventions were done to promote using CRS included face-to-face education, web/video-based education or written educational materials, distribution of free or subsidized CRSs, trained CRS technicians, and CRS installation checkpoints [21].

In Iran, there have been limited studies on the use of CSS [22–24], and no study has been conducted on the use of the booster. Based on what is seen among Iranian people, most of the children are unrestrained in cars and parents hold younger children on their lap in the front seat of the car. Moreover, booster production in factories is limited, and there were few of them in the market; but CSS for children under 4 is more common. A part of the Iranian culture is baby showers in which the family of the woman who becomes a mother for the first time provides all the necessary supplies for the baby. That is why there are special stores in the market that sell all infant supplies to customers. These include clothes, nursing bottles, prams, carriers, and even CRS for families who can afford them. In a survey, sellers responded that customers buy supplies more expensive than CRS for these showers, but the purchase of CRS is limited. Most of the CRS distributors were unaware of the existence of boosters.

These restrictions and barriers exist in Iran, but studies showed other barriers using CRSs. Common perceived barriers to correct restraint use include difficulty interpreting instructions and labels among parents; remembering and attending to correct use information; lack of information and behavioral feedback on how to correctly install and use a child restraint; and confidence in ability to install, use a child restraint correctly [25], special LMICs the cost of the child restraint, lack of knowledge and awareness of parents or caregivers and relatively low safety standard for vehicles [26]. In addition to the above special barriers to booster seats included fear of being teased, and wanting to feel and be seen as more mature by wearing a seatbelt only [27]. CRSs are considered as a mechanism to discipline children rather than a safety device by parents and as children became older they actively seek opportunities to negotiate the non-usage of restraints [28].

Therefore, the present study was conducted to investigate [1] the frequency of using children restrainesd system and [2] parents’ knowledge, and socio-demographic determinants toward CRS use for children under 10 years of age in Tabriz, Iran.

## Methods

### Study design and precedure

The present cross-sectional study was conducted on 700 children stated in cars. After observation children in cars whether restrained or not, socio-demographic information and parents’ knowledge were evaluated by questionnaires from July to August 2019 in Tabriz city, northwestern Iran.

### Sample size calculation

Sample size calculation was performed according to the study of Moradi M et al. [29], that reported a prevalence of 4.3% use of child safety seat in Tehran, d = 0.02 and confidence interval of 95%, the sample size was obtained equal to 630. Considering a 10% drop-out rate, a total of 700 cars were included.

### Participants and data collection

The present study was conducted on 700 cars that seated children under 10. The sampling precedure was multi-stage cluster sampling teqnique, followed by Martínez-Sánchez et al.’s method of direct in-field observation of drivers’ behaviors model [30]. To collecect data, the city of Tabriz was divided into three separate areas (high, moderate, and low) based on socioeconomic status. Then, from each area, two healthcare centers (in which children were brought for care or vaccination, including 10-day-old infants to children under 7 years of age), two primary schools (children aged 7–13 years), two parks (all children under the age of 10), and two kindergartens (children aged 3–6 years) were randomly selected by probability-based sampling. To avoid selection bias and social desirability bias, we gathered the information of using CRS with observation before questionnaire administration. Six trained observers conducted observations. Observation was perfomed with direct in-field observation method. Direct observation of driving behaviors may provide stronger and more valid documentation of actual driving behaviors than self-reported measurements; because self-reported measurements might stem for social desirability bias [31, 32]. The observers were MSc students working as traffic researchers who received three 60-min training sessions on how to complete the study questionnaire successfully. Their recordings on the first day of data collection were evaluated by one of the researchers in the same location to guarantee quality. Daylight hours of 7–12 a.m. and 2:00–5:30 p.m were chosen for observation because in these times observation is easier, and children were more likely to be observed. The observation was conducted in the morning and afternoon on both working days and holidays. In this method, observers stood at the parking space of the selected locations and observations were conducted as cars arrived at these places. They looked at children’s state inside the car, i.e., whether or not the child was restrained. After observation of children in cars whether restrained or not, questionnaires were administered for all of the parents. Following the observation, the purpose of the study was explained. Parents who were willing to participate completed the informed consent form and answered the questionnaires.

### Inclusion criteria

The inclusion criteria were cars which seated a child under 10 and one of the parents who was willing to participate in the study, and completing the questionnaires. In the current study, three-wheel, two-wheel, and heavy vehicles were excluded.

### Measurements

To determine the rate of CRS use, researchers observed the cars and completed a checklist (the use of CSS, booster, and seat belt). The correct way of installing the CRS was based on age (installation of CRS in the front or back seat of the car, and the correct installation of the CRS, forward-facing after 15 months or rear-facing before 15 months, based on the age and body size of the child). The back seat of the car is the safest place to install CRS. The installation of forward-facing CRS in the back seat of the car for children up 15 months, and rear-facing for children under 15 months provide the best protection [33].

For children over 4, the booster should be placed in the back seat of the car, and the seat belt should be adjusted so that it is away from the child’s neck area and across the shoulder, and places over the pelvis.

To assess the parents’ knowledge about and the barriers to CRS use, by a literature review and examining the status of the Iranian society, a questionnaire was developed. The questionnare consisted of two parts; first part included questions about general knowledge toward using CRS, and the second part consisted questions toward barriers of using CRS. The first part included two questions: "At what age can children wear the car seat belt?" and "By what age should a child sit in a CRS?" and the scoring was as follows: if neither of the two questions was answered, it indicated lack of knowledge; if only one of the two questions was answered correctly, it indicated moderate knowledge; and if both questions were answered correctly, it showed the parents' high level of knowledge. In the second part, parents were asked about barriers toward use of CRS with nine questions (e.g. lack of knowledge, lack of law and policy on CRS, high cost of CRS, lack of space in the car, parents' inattention to buy a CRS, needing a lot of time to restrain the child to the CRS, the child’s discomfort in the CRS, the child being to told not needing it, the child's reluctance to sit in its seat) which were answered with *yes* or *no*. The content validity of the questionnaire was qualitatively assessed by ten experts in the fields of traffic and children sciences. Some alterations and modifications were done afterward. The scores of content validity (CVI) were computed based on the simplicity, relevancy and clarification of each item. A CVI score of higher than 0.75 was considered as reasonable. Content validity ratio (CVR) scores were calculated based on the necessity of each item. A CVR score of equal to/higher than 0.59 was envisaged a good content validity by the experts. The mean of CVI and CVR was 0.83 and 0.78, respectively, signifying a good content validity for the scale. To assess the reliability, a pilot study conducted on 30 parents who didn’t include in the final sample. The Cronbach’s α was 0.77.

Underlying variable included car type, car crash history in the last two years (yes, no), type of crash (injured, damaging), driver’s relationship with the child (parents, non-parents), driver's gender (father, mother), child gender (boy, Daughter), the age of the child (based on year and month), the number of children under 10 in the family (one, two, three, four or more) and the age of the parents.

### Socio-demographic evaluation

A 6-item questionnaire was used to determine the socioeconomic status (SES), the validity and reliability of which have been confirmed in Iran. These 6 items include the job of the household head (the main source of income), the education level of the household head, the total monthly income of family members, the monetary value of the house, the monetary value of the private car, and the proportion of healthcare costs in total household expenditures. The SES classification was performed in such a way that a score less than 15.30 indicated low SES, scores 15.30–18.63 indicated moderate SES, and a score above 18.63 indicated high SES [34]. The study received ethical approval from the Ethics Committee of Tabriz University of Medical Sciences (Identifier: IR.TBZMED.REC.1397.1009). All methods of the current study were carried out in accordance with guidelines and regulations of the declaration of Helsinki.

### Data analysis

The data were analyzed in SPSS v. 25. Descriptive statistics were used to determine the mean and standard deviation in the quantitative data and the frequency and percentage of distribution in the qualitative data. To compare the mean and percentage of the two groups of CRS users and non-users, independent t-tests were run for quantitative variables, chi-square test for qualitative variables, and logistic regression to predict the most important determinants of CRS use.

## Results

The mean age of the participants was 33.24 ± 7.11 years, and most of them were in the 28–37-year age group. About half of the participants (55.3%, n = 387) were women, and the majority of them had a child under 10 years of age. Most of the time, the father was driving, and half of the participants had a damaging crash in the last two years (50.7%). More information about the participants' demographic characteristics is given in Table 1.

**Table 1:** Demographic characteristics of the participants

**Demographic characteristics **	**N (%)**
**Parent’s age (years)**	
18 -27	155 (22.1)
28 -37	332 (47.4)
38 -47	193 (27.5)
48 -57	20 (2.85)
**Parent’s sex**	
Male	313 (44.7)
Female	387 (55.3)
**Child’s sex**	
Boy	372 (53.1)
Girl	328 (46.9)
**Number of children under 10 in the family**	
1	459 (65.6)
2	217 (31)
3 or more	24 (3.4)
**Relationship with driver**	
The parent	700 (100)
Not the parent	0 (0)
**Driver**	
Father	493 (70.4)
Mother	207 (29.6)
**Type of crash**	
Damage	355 (50.7)
Injuries and fatality	46 (6.6)
None	299 (42.7)

Of the 700 observed cars with child passengers, 328 (46.9%) of the children were boys and 372 (53.1%) were girls. There were 133 (19.0%) children under 2, of whom only six (4.5%) children had CSS installed in the back seat and used it correctly. For 21 (15.8%) children, the CSS was installed in the back seat of the car but incorrectly. There were 261 children (37.3%) aged 2–4 years, of whom 44 (16.9%) children had the CSS installed in the back seat and used it correctly. Of 306 (43.7%) children over 4, two (0.6%) children had a booster installed in the back seat and used it. Overall, the safe behavior of CRS use was 6 (4.5%) among children aged under 2 years, was 44 (16.9%) among children aged 2–4 years, and was 0.6% among children aged over four years. Moreover, 229 children (32.7%) wore seat belts. More information on the frequency of CRS based on age is given in Table 2. The most important reasons for non-use of CRS were: a lack of laws and policies (59.7%), lack of knowledge (59.6%), the high cost of CRS (57.6%), the unwillingness of the child to sit in the CRS (46.4%), and needing a lot of time to restrain the child to the CRS (37.9%), respectively. A chi-square test was performed to assess the relationship between the parents' demographic variables and child seat use. Based on Table 3, there was a significant relationship between parents' knowledge of the benefits of CRS and its use (*p* < 0.001). There was also a significant relationship between the socioeconomic status of the household and the use of CRS (*p* < 0.001(. The findings showed that female drivers were more likely to use CRS (*p* < 0.001). However, there was no relationship between the driver's seat belt use and CRS use (*p* > 0.05). The results of the regression analysis showed that the most important predictors of CRS non-use are the child's age, parental knowledge, and the socioeconomic status of the household (Table 4).

**Table 2:** Frequency and percentage of CSS, booster and seat belt use based on age

	**Age group (years)**								
	<2			4-Feb			>4		
	No	Yes	Total	No	Yes	Total	No	Yes	Total
**CSS use**	99 (74.4)	34 (25.6)	133 (100)	208(79.7)	53(20.3)	261(100)	-	-	-
**Rear-facing**	6(4.5)			-	-	-	-	-	-
**Forward-facing**	21(15.8)			44 (16.9)			-	-	-
**Booster use**	-	-	-	-	-	-	304(99.4)	2(0.6)	306(100)
**Safe behavior**	6(4.5)			44(16.9)			2(0.6)		
**Seatbelt use**	115(86.5)	18(13.5)	133(100)	198(75.9)	63(24.1)	261(100)	158(51.6)	148(48.4)	306(100)
**Child sitting in the front seat**	84(63.2)	49(36.8)	133(100)	120(46)	141(54)	261(100)	94(30.7)	212(69.3)	306(100)
**Unsafe behavior**	43 (32.3)			110 (42.1)			140 (45.8)		

*CSS* Child safety seat

**Table 3:** The relationship between CRS use with parental awareness and drivers' demographic characteristics

	**CRS use**		***P*-value**
	**Yes**	**No**	
**Awareness of the age of wearing a seatbelt**			0.096
*Yes*	14 (24.1)	44 (75.9)	
*No*	100 (15.6)	542 (84.4)	
**Having information about CRS**			<0.001
*Yes*	99 (25.8)	284 (74.2)	
*No*	15 (4.7)	302 (95.3)	
**Awareness of the age of using CRS**			<0.001
*Yes*	51 (26.8)	139 (73.2)	
*No*	63 (12.4)	447 (87.6)	
**Child’s sex**			0.759
*Boy*	55 (16.8)	273 (83.2)	
*Girl*	59 (15.9)	313 (84.1)	
**Number of crashes in the past two years**			0.091
*0*	54 (18.1)	245 (81.9)	
*1*	19 (9.5)	181 (90.5)	
*2*	21(25)	63 (75)	
*3*	6 (12.5)	42 (87.5)	
*4 or more*	14 (20.3)	55 (79.7)	
**Type of crash**			0.311
*Damage*	51 (14.4)	304 (85.6)	
*Injuries*	7 (15.2)	39 (84.8)	
**Driver’s sex**			0.043
*Male*	71 (14.4)	422 (85.6)	
*Female*	43 (20.8)	164 (79.2)	
**Socioeconomic status**			<0.001
*Low *	13 (5.4)	227 (94.6)	
*Moderate*	34 (15.2)	189 (84.8)	
*High*	67 (28.3)	170 (71.7)	

*CRS* Children restrained system

**Table 4:** Barriers of CRS use based on the logistic regression model

**Variables**	**OR**	**CI (95%)**		*P*-value
		Min	max	
**Parent’s age**	1.039	0.999	1.081	0.057
**Parent’s gender **				
*Female *	1	-	-	-
*Male*	0.909	0.544	1.516	0.714
**Number of children under 10 in the family**	1.163	0.755	1.791	0.494
**Child’s sex**				
*Boy*	1	-	-	-
*Girl*	1.247	0.811	1.917	0.315
**Child’s age **				
*>4*	1	-	-	-
*<2*	0.742	0.122	4.518	<0.001
*4-Feb*	0.903	0.173	4.715	<0.001
**Driver’s sex **	1	-	-	-
*Female*				
*Male*	1.094	0.679	1.764	0.711
**Number of crashes**	0.947	0.801	1.12	0.523
**Type of crash**				
*None*	1	-	-	-
*Damage*	0.991	0.344	2.852	0.986
*Injuries*	1.036	0.415	2.587	0.94
**Knowledge **				
*High*	1	-	-	-
*Low*	3.513	1.156	10.677	<0.027
*Moderate*	2.237	0.738	6.783	0.015
**SES **	1	-	-	-
*High*				
*Low *	6.53	3.308	12.892	<0.001
*Moderate*	2.286	1.392	3.756	<0.001

*OR* Odd’s ratio, *CI* Confidence interval, *SES* Socioeconomic status

## Discussion

The present study assessed the knowledge, barriers, and use of CRS among parents of children under 10 years of age in Tabriz. The findings revealed a low rate of CRS use among households. The rate of CSS use was 15.1%, and the rate of booster use was 0.6%. However, in countries that have successfully reduced road traffic injuries (RTIs), such as Australia, the rate of CRS use in children is 90% [7]. This rate is 86% in the United States [35], 93% in Sweden [36], and 92% in Italy [37]. The rate in Iran was similar to the rate of Turkey (20%) [38] and China (10%) [6]. This difference in the use of CRS can be due to the fact that developed countries such as the United Kingdom, Australia, Sweden, and all US states have mandatory CRS use laws, but there is no mandatory law on CRS use in Asian countries such as China, Turkey, and Iran.

In the present study, in addition to the lack of law and policy, the most important reasons for not using CRS were a lack of knowledge, the high cost of CRS, the unwillingness of the child to sit in the CRS, and needing a lot of time to restrain the child to the CRS. Similar to the present study, other studies [23, 39] have reported the high cost of CRS as one of the barriers to its use. No all people in rich countries have a good economic status, but all children are restrained in cars. Different countries have used different strategies to ensure children's travel safety inside the car, including the government’s support or the payment of subsidies for CRS purchase [2].

The other obstacle to CRS use was that parents thought that the child had grown so old that he/she did not need CRS anymore, a result which was similar to the results of other studies [23, 39]. This suggests that parents are unaware of the use of boosters and the safe behaviors within the car for children. Therefore, it is necessary to offer direct and indirect training programs through various methods to improve safety inside the car. A study in Iran has shown that an educational program for children in kindergartens and primary schools is necessary to make them aware of the importance of using CRS in the car and also demand such a device (feeling the need to have this device and demand to provide it) [40].

Consistent with other studies [23, 39], the present study demonstrated that parents consider the intolerance of children for sitting in CRS as one of the barriers to its use. They also think that using CRS is not necessary, and holding the child on their lap is the best way to protect them [23]. All of these can be due to parents' lack of knowledge about the necessity of using CRS.

The findings of the present study showed that most parents had low knowledge about the age of using seat belts and CRS. The findings were consistent with those reported by Howard et al. who estimated that parents have moderate to poor knowledge [39]. Therefore, direct and indirect education regarding the protection of children inside the car is vital. Indirect education, in particular, can be effective. Even in Iranian films, movie stars sit their children in the front seat or leave them in the car without CRS. Increasing the information and knowledge of parents can increase the use of CRS. Based on the study by J W Lee et al., the implementation of educational programs about CRS through radio, television, and newspapers is effective in increasing parental information about CRS use in Latin communities [19]. Other studies have reported that educational interventions such as holding educational classes can increase parental knowledge and use of CRS [19, 39, 41]. Even some countries, such as the United Kingdom, have set up funds to educate children of different ages at school, equip Good Egg Safety stores and their staff to sell CRS, and have store clerks present some education [42]. The payment of subsidies, installment payments, or paying by checks, plus increasing parental awareness, can increase the use of CRS. Along with these programs, CRS producers must provide completely safe and standard equipment, produce more boosters, and advertise them.

Legislation and law is a key in CRS use [26]. The law and relative regulations for driving on the road concentrate on child protection in cars. For example, in Italy carrying children in trucks or vans is forbidden in Italy. The driver is responsible for children in cars. If the children aren’t restrained properly, the driver is punished and has to pay a fine between 74 and 300 euros, also loses 5 points for his/her driving license [43]. Then it is needed in Iran laws should be approved and implemented.

The law and relative regulations for driving on the road concentrate on child protection in cars. For example, in Italy carrying children in trucks or vans is forbidden in Italy. The driver is responsible for children in cars. If the children aren’t restrained properly, the driver is punished and has to pay a fine between 74 and 300 euros, Also loses 5 points for his/her driving license [43]. Then it is needed in Iran laws should be approved and implemented.

Despite the effectiveness of the CRS in preventing injuries, sometimes it is installed incorrectly, as was the case for 21 children (15.8%) under 2 in this study. In a study conducted in Italy, the rate of CRS use was 92%, but more than 30% of them were installed improperly [37]. In the United States, the rate of correct CRS use was reported to be 17% for school-aged children and 72% for infants [35]. In Turkey, the total use of CRS was 20%, and the rate of correct use was 10% [38]. Incorrect installation of CRS can be due to the parent’s insufficient information about it [38]. It may be necessary to provide training in educational programs on how to install CRS properly. At the other hand cars are needed to be equipped with at least two seating positions with ISOFIX, of which at least 2 need to be equipped with Top Tether. The car manufacturer needs to check the available space for rear-facing ISOFIX CRS, forward-facing CRS, and booster seat fixtures [43].

Consistent with other studies [6, 7, 38], the findings of our study also reported a significant relationship between parental knowledge of the benefits of CRS and its use. Studies conducted in China, Australia, and Turkey [6, 7, 38] have shown that the socioeconomic status of the household is related to the use of CRS. The present study also found a significant relationship between the two; college-educated drivers were more likely to use CRS than low-educated parents. This link can be attributed to the higher knowledge of parents which helps them better understand the risk of traffic crashes for children, and this increases the use of CRS [6]. There was also a significant relationship between the monthly household income level and the use of CRS; as the family's income increased, the use of CRS increased. Increasing income may increase access to CRS and purchasing power. Thus, low-income families should receive more attention. The results of R Apsler et al.'s study showed that providing free CRS to low-income families who attend training classes significantly increases the use of CRS by them, without the need for other interventions [14]. Another study showed that lowering the price of boosters through discount programs is an effective factor for increasing the use of boosters by low-income families [19]. In Iran, the allocation of government subsidies to low-income families for buying CRS can encourage them to use it.

Our results revealed that there was no association between the driver’s use of seat belts and the use of CRS, which was consistent with other studies [6, 7]. However, a study in Turkey showed that the use of CRS is significantly affected by the driver's use of seat belts [38]. More research is required and other factors must be considered in this regard. Based on our findings, female drivers used CRS for their children more than male drivers, which is consistent with other studies [6]. This relationship can be due to maternal love and the greater caution of mothers in caring for their children. However, the studies conducted in Australia and Turkey [7, 38] did not report a link between the driver’s sex and CRS use. The results of the regression analysis indicated that variables such as the child's age, parental knowledge, high education level, and income can affect the use of CRS. Sometimes parents think that their child is old enough to not use CRS, or that CRS is for younger children [44]. All these cases show a lack of awareness because the use of CRS increases with increasing the level of education. Safety experts recommend the use of boosters for children over 4 [18]. The role of the booster is lifting children and teens to improve the performance of the seat belts and thus decrease serious injuries by 59% [17]. It is concluded that education on CRS use is essential for parents. Studies have also shown that the media and educational campaigns are useful tools for increasing parent’s information about boosters [19, 45]. Of course, at the same time as educational programs, changes should be made at the environmental level such as having access to safety seats and implementing rules of using safety seats and not allowing children to sit in the front seat. Ecological approaches can be used in this field. Ecological approaches examine health-related issues by a multi-level interventions considering interpersonal, social, environmental, organizational and political levels. In interpersonal level implemented educational programs [46, 47]. Accessing to safety seats is placed in environmental system. Having proper isofix system related to organization system and implementing rules of using safety seats and not allowing children to sit in the front seat connect to policy level. Studies have revealed interventions at different levels of ecological approach could cause synergistic effects of the desired behavior [48]. Intervening at a particular level without considering other levels may waste resources and time. One of the most important ways to decrease road crash death and injuries is the safe system. The safe system concentrates to safe road transport. The aim of Safe System is to introduce system which eliminates deaths and serious injuries [49]. In Iran, both roads and vehicles need to be made safe. Also, the rules should be reviewed and implemented, and safe traffic behaviors should be taught to all users. One of the strong points of the present work was that the real behaviors of the children in real environments were recorded exactly. The observers stood at parking lots that they could observe whether children restrained or not. Then They wanted to patents or child giver to answer the questionnaire; thus, the results of this study were more real than those of the self-reporting studies. This study was the first observational study conducted in the northwestern part of Iran, in which the children restrained behaviors were investigated objectively. One of the limitations of the present study was that, we assessed parents' knowledge with two questions. We didn’t survey if they knew about the age child being in a rear-facing child restraint or using the booster seat. Moreover, the observers were likely to make mistakes in terms of recording the approximate age, high and weight of the children; age, weight and height was recorded only based on their features.

## Conclusions

According to the results of the current study, the frequency of CRS use and parents knowledge toward CRS use was low. The parents with higher education and those with higher socioeconomic status had higher rate of CRS use. Based on the low rate of CRS use and poor parental knowledge about it, education of parents toward boosters use and benefits of using CRS, enforcing mandatory laws and ploicies for CRS use in Iran, and allocation of government subsidies to low-income families for purchasing CRS are suggeted as essential strategies to increase CRS use.

## Supplementary Information

**Additional file 1.**

## Data Availability

The data collection tools and datasets generated and/or analyzed during the current study are available from the corresponding author on reasonable request.

## Abbreviations

CRS: Child restraint system
CSS: Child safety seat
MVC: Motor vehicle crashes
RTI: Road traffic injuries
CVR: Content validity ratio
CVI: Content validity index
SES: Socioeconomic Status
LMICs: Low and low middle income countries
